# Continuous Preparation and Properties of Silica/Rubber Composite Using Serial Modular Mixing

**DOI:** 10.3390/ma12193118

**Published:** 2019-09-25

**Authors:** Lin Zhu, Yiren Pan, Xiaolong Tian, Huaqiao Liu, Huiguang Bian, Chuansheng Wang

**Affiliations:** School of Mechatronics Engineering, Qingdao University of Science and Technology, Qingdao 266061, China; qustzhulin@163.com (L.Z.); qusttxl@126.com (X.T.); qustlhq@126.com (H.L.)

**Keywords:** serial modular, continuous mixing, silica/rubber composite, silanization reaction, preparation and properties

## Abstract

In order to efficiently prepare high-performance silica/rubber composites for use in the tread of semi-steel radial tires, a serial modular continuous mixer was designed according to the principle of modular functionalization. The modular structure and serial process helped control the accuracy of the silanization reaction. Synchronous four-wing serrated rotors and reverse meshing reaction mixing twin-rotors utilized shear flow and elongation flow to improve the dispersion. In this paper, the mechanism of serial modular continuous mixing was analyzed, and the influence of the core reaction mixing zone (various mixing elements) on silica-filled compounds was investigated by cooling visualization experiments, including dispersion, and the silanization reaction degree. Meanwhile, a comparative experiment between serial mixing and two-stage mixing was conducted, which showed that the serial process comprehensively improved the dispersion, mechanical properties, and dynamic mechanical properties of silica/rubber vulcanizate.

## 1. Introduction

In recent years, silica has been widely used as a reinforcement agent in the tread rubber of semi-steel radial tires because it improves the mechanical performance, reduces the rolling resistance, enhances the wet skid resistance, and shortens the braking distance [1,2,3,4,5,6]. Thus, methods to prepare silica/rubber composites have received considerable attention. Previous studies have shown that silica is incompatible with most nonpolar rubbers due to the presence of significant numbers of silanol groups (Si–OH) in silica. Bifunctional organosilanes must be used as coupling agents to improve compatibility via chemical reactions [7,8,9,10,11,12,13,14], and a considerable amount of literature has been published on the optimal reaction conditions. These studies have shown that the reaction must be maintained between 145 °C and 155 °C for 2–3 min, and volatile products such as ethanol and water should be quickly eliminated [15,16,17,18,19,20,21]. Current preparation methods, such as batch mixing, serial mixing, and continuous mixing, are difficult to continuously prepare high-performance silica/rubber composites.

Currently, industrial silica/rubber composites are generally prepared by batch mixing. The high shear effect provided by rotors reduces the viscosity of the mixture and disperses the filler in a raw rubber matrix [22,23,24,25,26]. A major problem with this method is that significant heat is produced by the rubber because of the high shear, which generates temperatures much higher than the silanization reaction can tolerate. Therefore, “multi-stage mixing” (i.e., master mixing, drop, cooling, remixing, drop, cooling, remixing, etc.) has been used to develop a mixing method compatible with the silanization reaction. Although the performance has reached an acceptable standard, it has come at the expense of energy and production efficiency [27,28,29,30,31,32,33]. In addition, the tandem mixer invented by Peter et al. [34] consists of two mixers, where the upper one is responsible for initial mixing, and the lower intermeshing mixer carries out the low-filling supplementary mixing, which prolongs the reaction mixing time [35,36]. However, continuous production has not been achieved. Furthermore, the existing rubber continuous mixing equipment, such as the FCM (Farrel continuous mixer), Buss Kneader, have not been widely used in industrial processes because they can only process powdered or granular rubbers, which increases production costs. The proportion of raw rubber and filler and the uniformity of the compounds are difficult to guarantee [37,38,39,40,41].

In this article, a serial modular continuous mixer based on the principle of modular functionalization was introduced, which is more effective for silica/rubber composite preparation. The modular design ensures the accuracy of the ratio, and also has a good residence time, temperature control, and exhaust. At the same time, the continuous process also improves the production efficiency and reduces energy losses. The core components are the initial mixing rotors and the core reaction mixing twin rotors. Grace’s theory was used to guide their design, i.e., flows with strong elongational components are more efficient at mixing over a wider range of viscosity ratios [42,43]. The special geometry allows these components to produce elongational forces (except shear stress) to the compound, which enhances the dispersion and reaction extent. In this work, changes in the silica/rubber composite in the core reaction mixing zone were investigated by cooling visualization experiments. The mixing effect of serial continuous mixing, including the dispersion, physical and mechanical properties, and dynamic mechanical properties, was comprehensively investigated by comparing it with a batch mixing process.

## 2. Materials and Methods

### 2.1. Materials and Formulas

**Figure 1:**
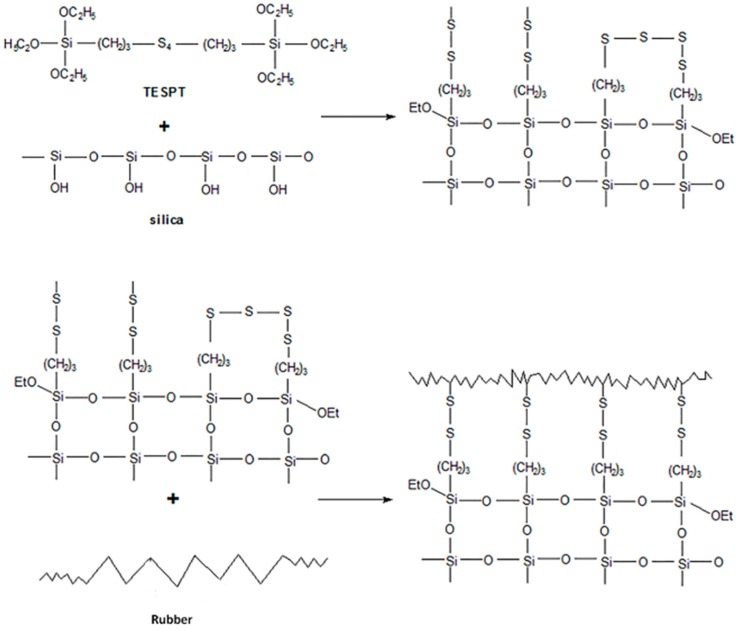
Silanization process between silica, a silane coupling agent (TESPE), and rubber.

The semi-steel tread formula in Table 1 was used to prepare high performance silica/rubber composites. SSBR (solution styrene butadiene rubber) and BR (butadiene rubber) were used as the matrix rubber. 45 phr silica and 25 phr carbon black were added as reinforcements. The silanization reaction between silica, a silane coupling agent (TESPE), and rubber is shown in Figure 1.

### 2.2. Equipment

**Figure 2:**
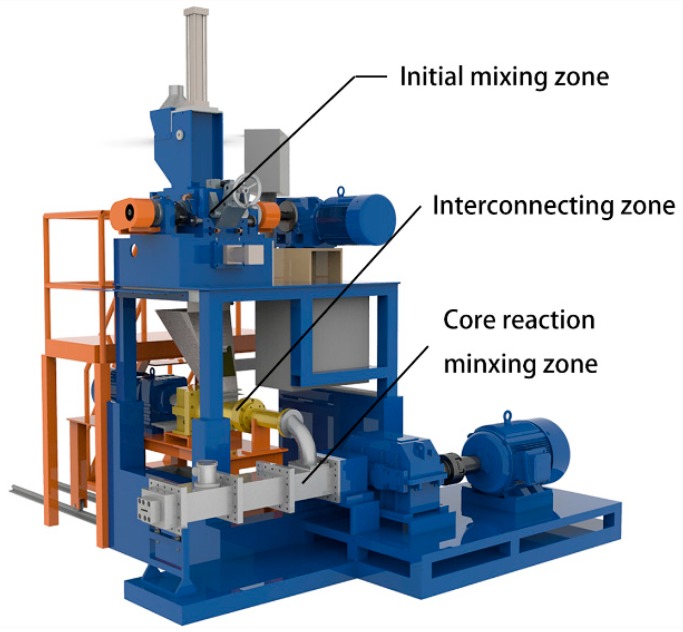
Integral structure of the serial modular continuous mixer.

A serial modular continuous mixer (model SCM 45/60) is a novel piece of equipment invented by Wang for continuous mixing of rubber. A schematic diagram of the SCM is shown in Figure 2. It is mainly composed of an initial mixing zone, an interconnecting zone, and a core reaction mixing zone, which are all arranged in-series. The rubber and filler successively pass through three zones to ensure complete dispersion mixing and reaction mixing. The composite material is finally continuously extruded from the head of the core reaction mixing zone.

**Figure 3:**
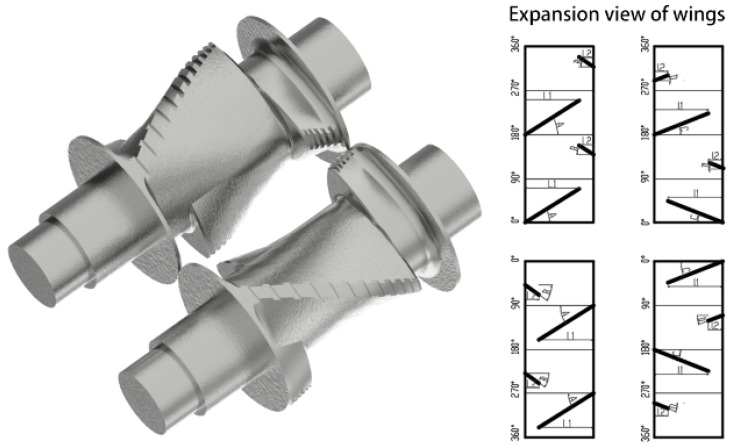
Structure diagram of synchronous four-wing serrated rotors.

Since the initial mixing zone is responsible for crushing, fusion, and distribution mixing, the synchronous four-wing serrated rotors shown in Figure 3 were designed. A long wing and a short wing are located at the same end of the rotor, with 135° between them. The other long wing (short wing) is at the other end of the rotor, symmetrical to the first (180°). The mixture is forced to move axially from one end of the rotor to the other and then back under the action of the opposite wings, which rapidly improves the uniformity of the composite. There are some grooves of equal width on the four wings, and the density increases with the circumferential flow velocity. These grooves are set up to increase the leakage flow and thus improve the uniformity of the composite.

**Figure 4:**
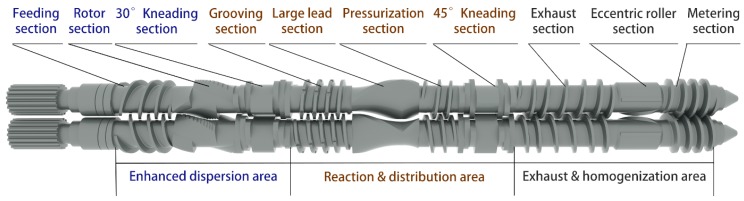
3D modeling diagram of the reverse meshing reaction mixing twin rotors.

The key component of the core reaction mixing zone is the reverse meshing reaction mixing twin rotors shown in Figure 4. These twin rotors are specially designed according to the characteristics of the silane mixing reaction, and help achieve simultaneous dispersion, reaction, exhaust, and homogenization. They also consist of an enhanced dispersion area, a reaction and distribution area, and an exhaust and homogenization area, with 10 different mixing elements. The feeding section, grooving section, pressurization section, exhaust section, and metering section are all variable lead threads to ensure the positive movement of the rubber. The difference between the rotor section and synchronous four-wing serrated rotor is that there are no grooves on the short wings. This design enhances the reverse transport capacity of rotor sections, so that the axial reciprocating circulation flows of compounds are formed in this position to improve the distribution effect. The kneading section ensures dispersion, distribution, and transport capacity, and the intensity of each is affected by the thickness of the kneading block and staggered angle. The large lead thread section is composed of forward and reverse double-head threads with a length ratio of 3:1, which can mix and control the temperature of the compounds. The main body of the eccentric roller section is a roller with an eccentric moment of 1.25 mm. There is a positive rotation wing and two straight wings on the surface of the roller, which separates the surface of the roller into three cavities. When the component operates, it places a strong tensile force on the compound and improves the dispersion and distribution effect.

The internal mixer (model GK 45E) is a meshing mixer produced by China Chemical Equipment Company. It is equipped with a PES3 meshing rotor, which is mainly used to prepare radial tires and high-quality rubber products. Other devices are listed in Table 2.

### 2.3. Sample Preparation

#### 2.3.1. Cooling Visualization Experiment

**Figure 5:**
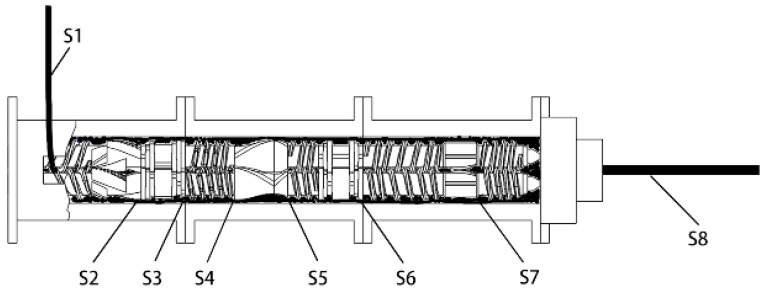
Schematic diagram of sample position.

According to the serial continuous mixing process shown in Table 3, a masterbatch was prepared. When the head stably extruded the mixture for 2 min, the mixer was stopped and quickly cooled, and then the cylinder of the core reaction mixing zone was removed. Samples S1–S8 were obtained from the positions shown in Figure 5.

#### 2.3.2. Comparison Experiment

Sample S9 was prepared by the internal mixer in two mixing stages. The temperature of the mixer body and rotors was set to 40 °C, and the rotor speed was 40 rpm. A fill factor of 0.7 was used. The masterbatch was prepared via the following method:

(i) Add rubber and mix for 30 s.
(ii) Add carbon black, 1/2 silica, and the rest additives, then mix for 30 s.
(iii) Add the rest of the silica, and mix to 110 °C.
(iv) Sweep and add oil.
(v) When the temperature reaches 125 °C and 140 °C, up and down the ram.
(vi) Mix to 155 °C and then drop.

The second stage involves placing the masterbatch into the mixer for remixing. The ram is raised and lowered every 30 s and drop at 150 °C.

The vulcanization system was added on an open mill, and then the vulcanizate was prepared by a flat-panel vulcanizer at 150 °C/10 MPa × T90 (min). The properties of samples S8 and S9 were characterized and compared, including filler dispersion, physical and mechanical properties, and dynamic mechanical properties.

## 3. Results and Discussion

### 3.1. Mechanism of Serial Modular Continuous Mixing

**Figure 6:**
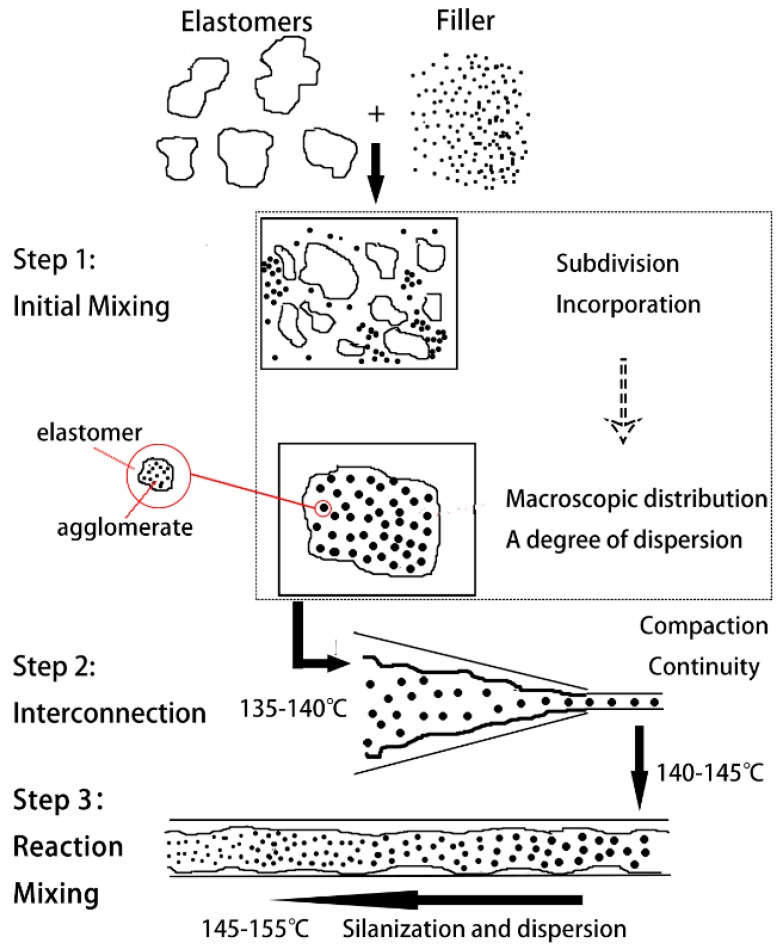
Mixing mechanism and process of serial modular continuous mixing.

The mechanism and process of serial continuous mixing were analyzed by combining rubber mixing theory and silanization reaction principles. The schematic diagram is shown in Figure 6. The entire mixing process was completed in three functional areas: The initial mixing zone, the interconnecting zone, and the core reaction mixing zone. The purpose of each functional area is clear, which is conducive to the optimal design of the core components and process design, to achieve a better completion effect.

The initial mixing zone was responsible for the incorporation and macroscopic distribution of rubber and filler. The structure was similar to a traditional mixer, and could accommodate materials in any kind of trade form and ensures the accuracy of the mixing ratio. However, there were some differences. (1) There was no need to carry out the silanization reaction in the initial mixing zone. The compound was immediately dropped when the temperature reached about 135–140 °C. Therefore, there was no problem in traditional mixing, i.e., it was difficult to maintain the rubber temperature in the range of the reaction temperature. (2) The initial mixing had a higher rotation speed (65 rpm) and a higher cooling water temperature (55 °C), which were more efficient than traditional mixing. (3) For the initial mixing rotors, fast feeding, rapid incorporation, and homogenization were more important, so the synchronous four-wing serrated rotors in Figure 3 were designed. Just like synchronous rotors, the synchronous four-wing serrated rotors had fast feeding, good consistency between batches, and no material jams (a specific analysis can be found in the doctoral thesis of Wang [44]). In addition, a number of variable depth grooves were arranged on the wings, which can rapidly incorporate the filler and improve the uniformity of the compound. At the beginning of mixing, the Mooney viscosity of the compound is high. When it is forced to pass through the gap between the rotor tip and mixer chamber wall, or between the two rotors, there will be a strong additional shear and elongation effect at the grooves, causing the compound to break and rapidly heat up. The resulting fresh surface helps incorporate filler. As the mixing proceeds, the Mooney viscosity decreases. When the viscosity of the compound reaches a certain value, the grooves can no longer produce strong shear forces, but the leakage flow increases, which helps improve the compound uniformity.

The second functional area—the interconnection zone, with a Y-shaped conical twin-screw as the core component—was responsible for catching the master compound and continuously, quantitatively supplying the downstream core reactive mixing zone. The temperature of the compound was maintained between 140 °C and 145 °C, and it was compressed to improve its compactness.

The core reaction mixing zone was the main component of dispersive and reactive mixing, which had the following characteristics. (1) Sufficient residence time due to the large length-to-diameter ratio; (2) strong ability to control the reaction temperature from the independent temperature control of the segmented cylinder and drilling cooling; (3) high dispersion ability from the reverse meshing reaction of the mixing double rotors; and (4) effective removal of volatile reactants via the vacuum exhaust hole and the changing rotor configuration. Finally, after passing through the above three functional areas, the strip compound was continuously and stably extruded.

Compared with a traditional multistage process, the serial modular continuous mixing process was undoubtedly a more efficient and low-consumption way to prepare silica/rubber composites. It was not only suitable for the laboratory, but also for industrial-scale production.

### 3.2. Mixing Effect of Core Reaction Mixing Zone

#### 3.2.1. Filler Network (Payne Effect)

**Figure 7:**
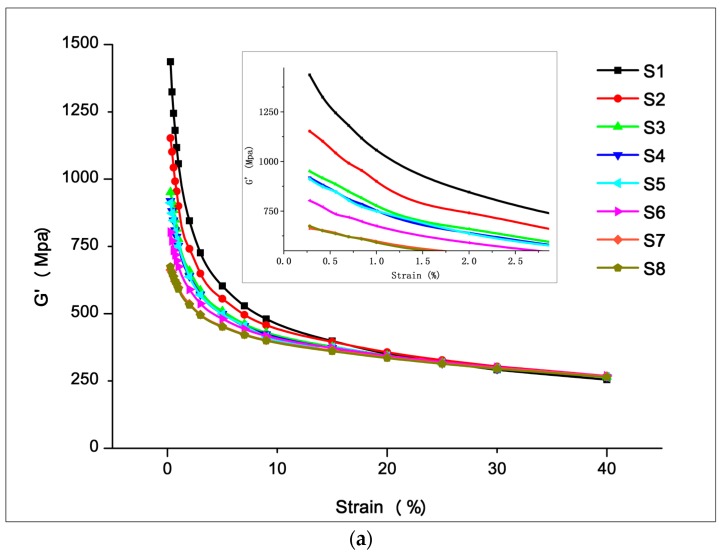

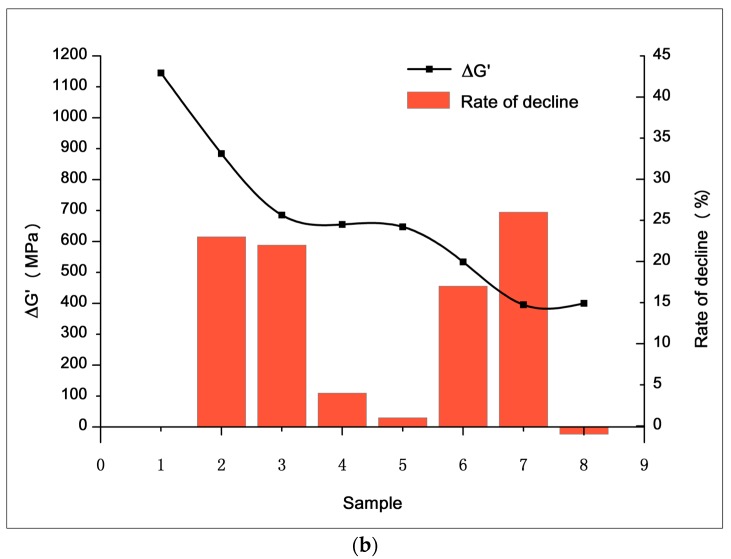
Result of Payne effect measurements. (**a**) The “G′-strain” curve of S1-S8, and (**b**) G′ difference and rate of decline of S1–S8.

The Payne effect is a particular feature of the stress–strain behavior of rubber, especially rubber compounds containing fillers. The effect is observed under cyclic loading with small strain amplitudes, and manifests as a dependence of the viscoelastic storage modulus on the amplitude of the applied strain [45]. With a rubber process analyzer, the Payne effect of the samples was assessed by measuring the storage modulus (*G*′) when the strain increased from its lowest to its highest value (0.28%–40%) at a constant frequency (1 Hz) and temperature (60 °C). The storage modulus (*G*′) variation with the strain amplitude of the silica/SSBR compounds, as shown in Figure 7a, was used to analyze the filler-network structure. The results in terms of Δ*G*′ and rate of decline are given in Figure 7b. Some trends were visible from the Payne effect measurements.

The decreasing rates of S1–S2 and S2–S3 were 23% and 22%, respectively, indicating that the rotor section and 30° kneading section effectively improved the dispersion of the filler. In the rotor section, axial spiraling flow and circumferential "∞" flow of the rubber occurred, accompanied by strong shear and tensile forces. Thus, a better dispersion mixing effect was obtained. The 30° kneading section was composed of 6 thin, two-end kneading discs. The staggered angles were positive 30°/30°/90°/90°/30°/30° in turn. The design balances the mixing capacity and the conveying capacity, so that the kneading block could squeeze, shear, and transport the rubber forward. The experimental results show that the mixing element effectively promoted the dispersion of filler.

The decreasing rates of S3–S4 and S4–S5 were 4% and 1%, respectively, showing that the dispersion was not improved after passing the grooving section and large-lead screw section. This was because there were no strong shear forces to break the filler agglomerates. The significance of setting up these two mixing elements lies in the following two aspects. (i) Distributive mixing. The six grooves on the grooving section increased the leakage flow, which facilitated the exchange and mixing of materials before and after. Several wedges formed between the large-lead screw section and the inner wall of the barrel. When rotated, the composite was forced to flow over a wide area in a circular direction. At the same time, the conflict between the forward and reverse threads caused the compound to move back and forth in the axial direction. The resulting push–pull effect, cross flow, and convolution deformation helped promote distributive mixing. (ii) Temperature control of the composite. In these two sections, the larger contact area between the rubber and the barrel helped stabilize the temperature of the rubber in the reaction range via heat conduction.

**Figure 8:**
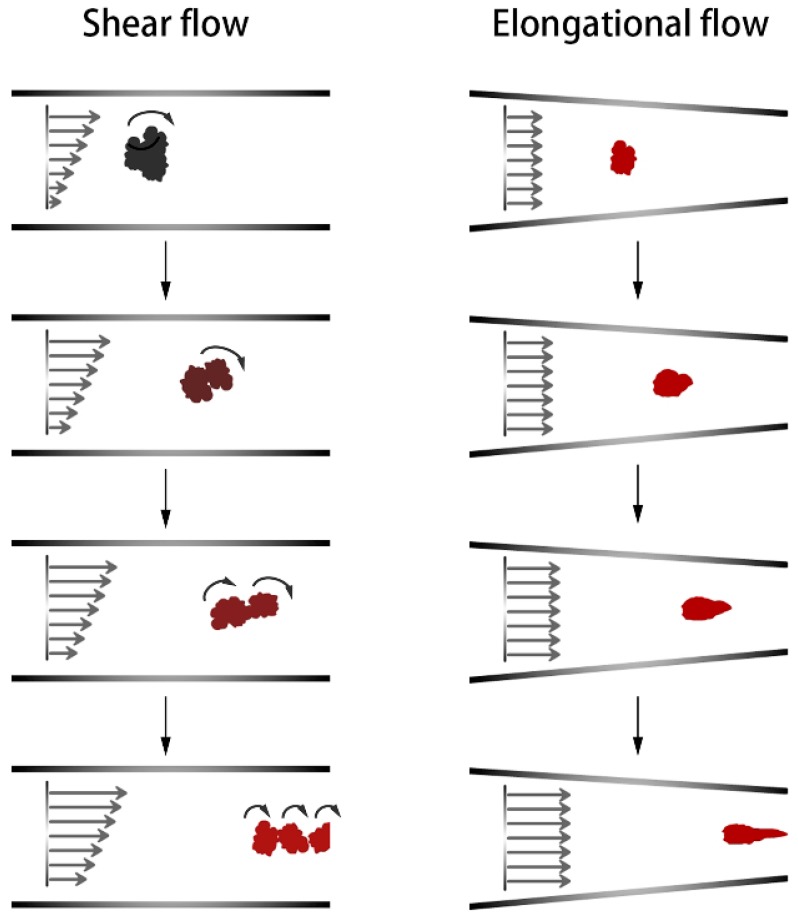
Schematic diagram of the effect of shear and elongation flow on filler agglomerates during the later mixing stage.

Another rapid increase in the filler dispersion occurred in the S5–S6–S7 stage. The 45° kneading section reduced the Payne effect by 17%, and the dispersion ability of the combined kneading block was once again demonstrated. A greater improvement occurred in the eccentric roller section, where the Payne effect decreased by 26%. The difference occurred because each of the two components had a different mechanical effect on the rubber. The kneading block element mainly broke the filler agglomerates by shearing. When the eccentric roller section rotated, the volume of the cavities between the component and the barrel, as well as the volume of the cavities between the positive rotation wing and straight wings changes. This changes periodically to form a strong tensile flow field, which promotes dispersion. The experimental results showed that shear and elongation flow effectively promoted the dispersion of filler. However, at the later stage of mixing, the elongation effect was better. Figure 8 shows that the influence of shear and elongation flow on agglomerates at a later period of mixing. At this stage, the compound showed a high viscosity melt state. Small agglomerates were encased in compounds and were difficult to break under shear forces. Shear forces may only cause the agglomerates to roll. At this point, the elongation flow effectively improved dispersion, and it deformed the filler agglomerates to form fresh surfaces. The contact area between the filler and rubber increased, which improved the dispersion. The experiment and analysis showed that adding a tensile element during the later stage of mixing helped improve filler dispersion.

It is worth noting that the Payne effect increased by 1% during the S7–S8 stage, i.e., the dispersion slightly decreased after the metering section and the head. This may have occurred because the compression of the head improved the compactness of the compound, which promoted the secondary agglomerates between the filler and filler, which increased the binding force.

#### 3.2.2. Degree of Silanization Reaction

**Figure 9:**
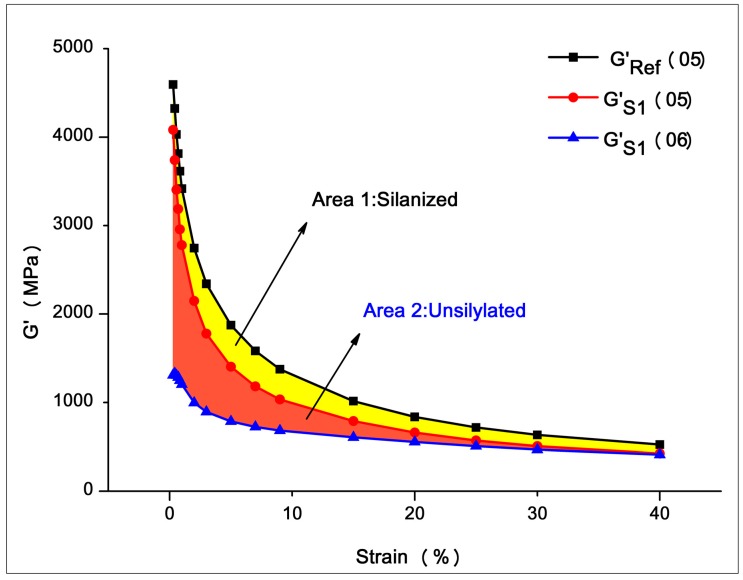
Test principle of the silanization reaction degree.

With a rubber process analyzer (RPA), the degree of silanization was assessed using the method shown in Table 4 [46,47]. Stage 1 involved preheating the sample, and Stages 2 and 3 broke the filler agglomerates caused by non-dispersion. In Stage 4, the samples were held at 160 °C, which intensified the polarity Brownian motion, and caused secondary agglomerates of the non-silanized silica to form. Stage 5 breaks the agglomerates of the non-silanized filler and $G_{S}^{\prime }\left(05\right)$ decreased. During Stage 6, nearly all filler networks were broken. During the experiment, a reference value $G_{Ref}^{\prime }\left(05\right)$ should be set, i.e., samples with the same formula without the coupling agent should be tested. Since there is no silanization reaction, the filler agglomerates were the largest, and $G_{Ref}^{\prime }\left(05\right)$ decreased the most, as shown in Figure 9. According to the analysis of the figure, the difference between curve $G_{Ref}^{\prime }\left(05\right)$ and curve $G_{S}^{\prime }\left(05\right)$ was caused by the partial silanization of samples, and the overlap of curve $G_{S}^{\prime }\left(06\right)$ represents the state when samples reached maximum silanization. The formula to characterize the degree of the silanization reaction is as follows (note: This formula is only applicable to comparisons made between samples with the same formula):

(1) $$X=\frac{Silanized}{Maximum\;silanization}\;=\frac{Area\;1}{Area+Area\;2}\times 100\%\;=\frac{\int {G^{\prime }}_{Ref}\left(05\right)-\int {G^{\prime }}_{S}\left(05\right)}{\int {G^{\prime }}_{Ref}\left(05\right)-\int {G^{\prime }}_{S}\left(06\right)}\times 100\%.$$

**Figure 10:**
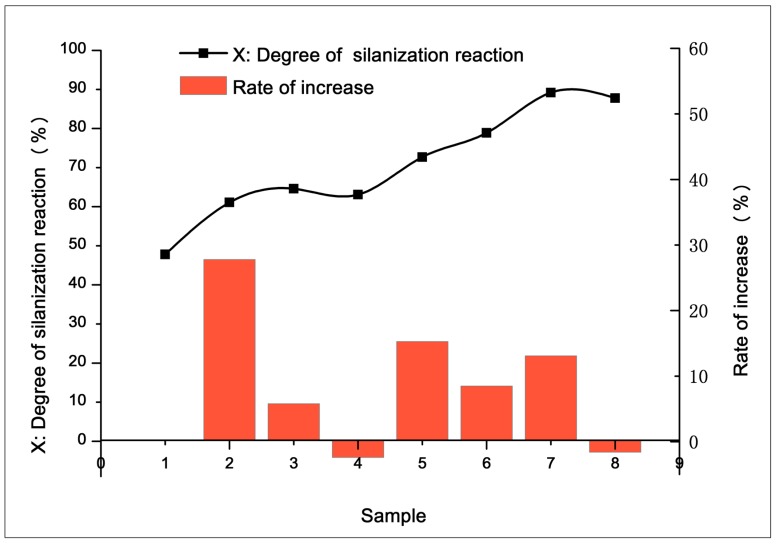
Result of silanization reaction degree measurements.

S1–S8 were tested to calculate the index of silanization degree *X* and the rate of increase during each stage, which are shown in Figure 10. Through analysis, the following trends were obtained:

(1) As the compound advanced through the core reaction mixing zone, the silanization reaction degree increased before remaining steady at a higher level. To some extent, the core reaction mixing zone promoted the silanization reaction.
(2) Silanization did not occur continuously and stably in the core mixing zone, and was affected by the mixing element.

In stage S1–S2, the rate of increase reached 27.8%, which indicated that the silanization reaction rate was fast when passing through the rotor section. This may be because the initial compound was dispersed to a certain extent. The silica and coupling agent were in contact with each other. When passing through the rotor section, the temperature reached about 145–150 °C, and the reaction started.

A slight decline (−2.4%) occurred during stage S3–S4, which might have been due to the axial blend of compound caused by the leakage flow of the grooved section. However, this helped improve the uniformity of the compound.

The reaction speed was fast when it proceeded through the large lead screw section, 45° kneading section, and eccentric roller section. The silanization degree increased by 15.3%, 8.5%, and 13.1% in these sections, respectively, which indicates that the silanization reaction temperature was appropriately controlled in these areas.

The degree of silanization reaction decreased during the S7–S8 stage (−1.6%), which might also be because the head pressure promoted secondary filler agglomerates, which degraded the weak interaction between silica and the compound.

### 3.3. Comparison of Vulcanization Properties: Serial Mixing and Two-Stage Mixing

The vulcanization properties of the compound prepared by serial continuous mixing were compared with those prepared by traditional two-stage mixing.

#### 3.3.1. Filler Dispersion

**Figure 11:**
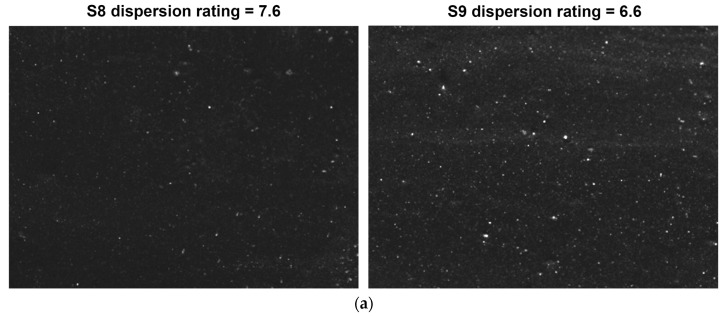

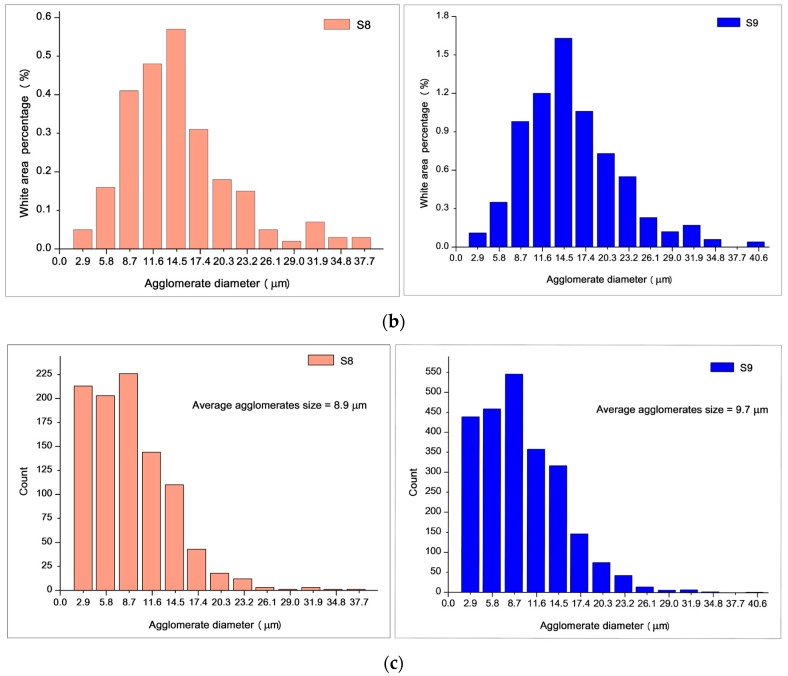
Results of filler dispersion tests. (**a**) Dispersion rating of S8 and S9, (**b**) histogram of white area percentage for S8 and S9, (**c**) histogram of agglomerates for S8 and S9.

Filler dispersion was tested using a DisperGRADER dispersion tester, which automatically obtained sample dispersion ratings according to ISO 11345 and ASTM D7723 standards. Figure 11 shows the test results of S8 and S9 (100 times larger), which indicate that (1) the dispersion rating of S8 was larger than S9, (2) the total percentage of the white area (2.5%) was significantly smaller than that of S9 (7.2%), (3) the white area of S8 contained a larger distribution of smaller particles (<20 μm), and (4) the average particle size of S8 was smaller (8.9 < 9.7). These results show that serial continuous mixing had a better dispersion effect on the silica/rubber composite, and that the mixing method combining shear and elongation flow was better.

#### 3.3.2. Mechanical and Machining Properties

The tensile strength and tear strength were measured using a U-Can TS2005b universal testing machine according to ASTM D412 and ASTMD 624. Hardness was measured using an indentation hardness tester according to ASTM D2240–75. The Mooney viscosity was measured with a U-Can Mooney viscometer according to GB/T 1232.1-2016. All test results are shown in Table 5.

After analysis, it was concluded that: (1) since the formula was unchanged, the hardness of S8 and S9 were nearly identical, which is reasonable. (2) The 100% modulus, 300% modulus, M300/M100, tensile strength, and tear strength of S8 were higher than those of S9 because the dispersion and silanization reaction degree of the compound formed by serial continuous mixing were better, which led to more bonding rubber content. The rubber had better resistance to deformation and damage because of the larger binding force between filler and rubber, as well as less filler agglomerates network. (3) In terms of the Mooney viscosity, S8 had better machining properties, which may be related to the smaller size and quantity of filler agglomerates.

#### 3.3.3. Dynamic Mechanical Properties and Abrasion

Dynamic mechanical properties of compounds were measured with a METTLER TOLEDO DMA (dynamic mechanical analysis). Tests were conducted using a frequency of 10 Hz, a 5N stress, 20 μm displacement, a temperature range of −60–60 °C, and a ramp rate of 2 K/min in plane shear mode. DIN abrasion (determining abrasion) was tested using a GT-2012-D DIN abrasion machine according to GB/T 9867-2008 standard.

**Figure 12:**
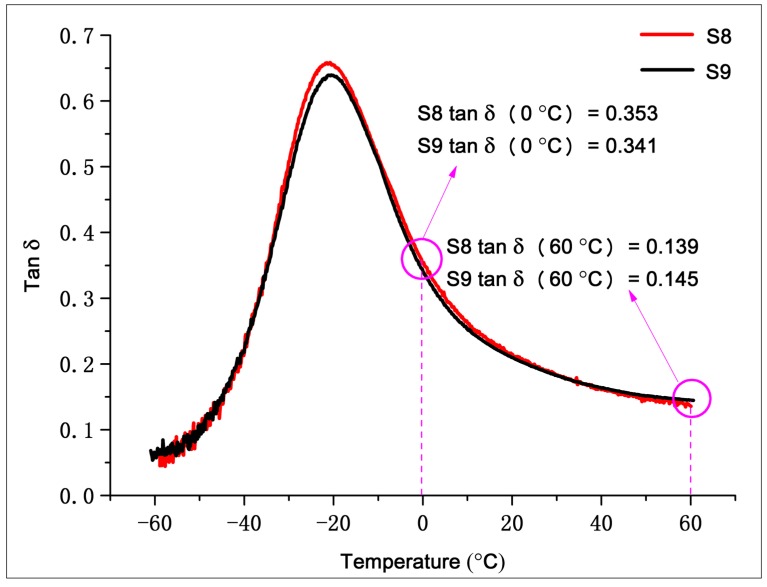
Results of dynamic mechanical property measurements.

The DMA test results of S8 and S9 in Figure 12 show that: (1) The Tg of S8 and S9 were consistent since they essentially had the same formula. (2) The peak value of tan δ for S8 was higher than S9 because the dispersion of the compound obtained by continuous serial mixing was better, and the occluded rubber content between silica agglomerate was lower. Therefore, there were more rubber molecules involved in dynamic deformation during the glass transition, resulting in a high loss peak. (3) The tan δ of S8 at 0 °C was higher, indicating that S8 had a higher wet skid resistance than S9 because the bonded rubber of S8 was higher, which gave it a higher resistance to dynamic deformation. (4) The tan δ of S8 at 60 °C was lower than S9, indicating a lower rolling resistance. At 60 °C, the loss of the system mainly arose from the filler network and the breaking and reforming of weak interactions between filler and rubber. S8 had a better dispersion and silanization, indicating that the filler network was less dense, and the coupling force between silica and rubber was stronger. Therefore, the hysteresis loss under dynamic deformation was lower.

There is a famous “devil’s triangle” relationship between rolling resistance, wet skid resistance, and abrasion resistance. For fixed formulations, excellent mixing machines and processes can improve one or both properties while also reducing the other properties as little as possible. Although serial continuous mixing improves the moisture resistance and reduces rolling resistance, it does not break the magic triangle relationship. According to the DIN abrasion test results of S8 and S9, the mass abrasion rates were 9.09% and 8.69%, respectively. Therefore, the wear resistance of the two were basically the same. It can be seen that serial continuous mixing improved the rolling resistance and wet grip without negatively impacting the abrasion resistance, and hence the lifetime, of tires.

## 4. Conclusions

Serial modular continuous mixing equipment was specially designed to mix a silica/rubber composite with modularization and continuity. In this work, the mixing effect of silica/rubber composite using serial continuous mixing was explored by cooling visualization and comparative experiments. The results showed that:

(1) The core reaction mixing zone effectively improved the filler dispersion and promoted the continuous silanization reaction.
(2) Both shear mixing and tensile mixing elements played important roles in improving the dispersion, and the tensile mixing element further improved dispersion during the later mixing period. The synchronous four-wing serrated rotor, combined kneading block, and eccentric roller all had good dispersing abilities. Among them, the eccentric roller element was more suitable during later mixing.
(3) In general, the serial continuous mixing process suitably controlled the silanization temperature, and the reaction degree gradually increased. However, the grooving section had little effect on promoting the dispersion and reaction. Thus, distribution effect should be further verified.
(4) The performance of the mixture slightly decreased after passing through the head, which was related to secondary filler agglomerates and the breakage of the weak interactions between the rubber and filler.
(5) Compared with a traditional two-stage mixing process, the serial continuous mixing improved the filler dispersion, physical, and mechanical properties and dynamic mechanical properties to different degrees, and was more suitable for the mixing of filler. This was related to the filler network, silanization reaction degree, and bonded rubber content.

## 5. Patents

For more information about serial modular continuous mixing method, please refer to the Chinese invention patent: “A serial rubber continuous mixing method”, patent no. ZL201410648268.6.

## Figures and Tables

**Table 1:** Semi-steel tread formula.

Supplier	Category	Trade Name	Content (phr)
SINOPEC (Beijing, China)	Rubber	SSBR	96.25
	Rubber	BR	30
	Activator	Zinc Oxide	2
	Processing aids	Stearic Acid	2
SOLVAY (Brussels, Belgium)	Filler	Silica	45
CABOT (Boston, MA, USA)	Filler	CB N234	25
Nanjing Shuguang (Nanjing, China)	Coupling agent	TESPT(Rsi-B)	5.4
RheinChemie (Mannheim, Germany)	Antioxidant	Microcrystalline Wax	1.5
Hansen & Rosenthal (Hamburg, Germany)	Plasticizer	Aromatic Oil	3
Shandong Shangshun (Heze, China)	Antioxidant	DMPPD	2
	Accelerator	DPG	0.8
	Accelerator	CZ	1.8
	Vulcanizer	Sulfur	1.3

**Table 2:** Experimental equipment.

Equipment	Model	Manufacturer
Mill	XK-160E	Dalian Rubber and Plastics Machinery Co., Ltd. (Dalian, China)
Flat-Panel Vulcanizer	QLB-400X400X2	Qingdao Yadong Machinery Group Co., Ltd. (Qingdao, China)
Rubber Process Analyzer	RPA2000	ALPHA (Akron, OH, USA)
Dispersion Tester	Disper GRADER	
Universal Tester	TS2005b	U-CAN (Taiwan, China)
Mooney Viscometer	UM-2050	
Dynamic Mechanical Thermal Analyzer	DMA/SDTA861e	METTLER TOLEDO (Zurich, Switzerland)
Din Abrasion Tester	GT-2012-D	GOTECH (Dongguan, China)

**Table 3:** Serial continuous mixing process.

Zone	Parameter	Value	Process
Initial Mixing	Temperature Controller	55 °C	1. Add rubber, and mix for 15 s 2. Add carbon black, 1/2 silica, and the rest additives, then mix for 15 s 3. Add the rest of the silica, and mix to 110 °C 4. Sweep and add oil, then mix to 140 °C and drop 5. Sequentially enter the interconnection zone and the core reaction mixing zone
	rotor speed	50 rpm	
	Filling Factor	0.75	
Interconnection	Temperature Controller	140 °C	
	Double-cone screw speed	20 rpm	
Core Reaction Mixing	Temperature Controller 1	145 °C	
	Temperature Controller 2	140 °C	
	Temperature Controller 3	130 °C	
	Twin rotor speed	40 rpm	

**Table 4:** Rubber process analyzer method used to measure the degree of silanization reaction.

Stage	Frequency (Hz)	Temperature (°C)	Time (min)	Strain	Test Parameters
1	0.1	60	5	0.28%	-
2	1	60	-	0.28%–40%	G′02
3	1	60	-	0.28%–40%	G′03
4	0.1	60/160/160	0/2.5/5	0.28%	-
5	1	60	-	0.28%–40%	G′05
6	1	60	-	0.28%–40%	G′06

**Table 5:** Test results of mechanical properties and machining properties.

Sample	Hardness (Shore A)	M100 (MPa)	M300 (MPa)	M300/M100	Tensile Strength (MPa)	Tear Strength (kN/m)	Mooney Viscosity $\left(M_{L}^{100℃}(1+4)\right)$
S8	60	1.9	8.7	4.60	18.2	54.9	66.5
S9	61	1.8	7.9	4.39	16.3	50.3	75.8
